# Experiences of women on the use of Implanon NXT in Gauteng province, South Africa: A qualitative study

**DOI:** 10.4102/hsag.v29i0.2237

**Published:** 2024-02-29

**Authors:** Junior M. Ntimani, Moreoagae B. Randa

**Affiliations:** 1 Department of Nursing Science, School of Health Care Sciences, Sefako Makgatho Health Sciences University, Pretoria, South Africa; 2 Department of Public Health, School of Health Care Sciences, Sefako Makgatho Health Sciences University, Pretoria, South Africa

**Keywords:** contraceptive methods, early removal, experiences, Implanon NXT, South Africa, women

## Abstract

**Background:**

Maternal-related illnesses and pregnancy-related deaths could be averted using contraceptives, particularly long-acting methods because they improve overall access to family planning because of their benefits, such as fewer visits to the healthcare facility, higher effectiveness and value for money. The introduction of Implanon NXT in the South African health system raised great concern about the number of women who returned to healthcare facilities for its early removal within a few months of insertion.

**Aim:**

This article focussed on exploring the experiences of women regarding early removal of Implanon NXT insertion.

**Setting:**

The study was conducted in four selected clinics in Region E sub-district, city of Johannesburg.

**Methods:**

A qualitative exploratory approach was followed using semi-structured individual interviews to collect data. Data were analysed using the content analysis method.

**Results:**

The most cited reasons for its discontinuity were that women experienced unpleasant side effects such as amenorrhoea, excessive bleeding and headaches, whilst other women found it to be reliable and convenient.

**Conclusion:**

Contraceptive methods empower women to take control of their lives and to decide on the right time for them to have children. It is, therefore, important to equip women with relevant information about the contraceptive methods so that they can make informed decisions and choices.

**Contribution:**

The study calls for health promoters to periodically conduct information sessions with women as guided by the National Contraception Policy Guidelines to ensure up-to-date practice.

## Introduction

Sexual and Reproductive Health Rights are enshrined within the Constitution of the Republic of South Africa (Constitution 1996) to protect both men and women equally. This is because of the importance of reproductive rights of women, which require women to exercise their autonomy in the family planning activities, including choice of contraceptive methods (Constitution 1996; Sustainable Development Goals commissioned in 2015).

The 2001 National Contraception Policy Guidelines (updated in 2012), the *National Health Act*, 2003 as well as the National Adolescent Sexual and Reproductive Health and Rights Framework Strategy (2014–2019) recognise the challenges and health needs of women and adolescents, particularly family planning needs. These legislative developments are part of ensuring access to contraceptives as a human right. The World Health Organization (WHO) also posits that contraceptive services, including contraceptive information, are a human right for all as they are essential to health (WHO 2020). This is because, with access to contraceptives or otherwise, family planning allows people to attain their desired number of children and determine child spacing. Contraceptives also allow people to avoid unwanted or unintended pregnancies and lower maternal ill health and pregnancy-related deaths (Stover & Ross 2010).

The South African government through the Department of Health has made some strides in ensuring the consistent provision of contraceptive services in the country. The National Contraception and Fertility Planning Policy and Services Delivery Guidelines were developed to provide the National Contraceptive Guidelines in South Africa (Department of Health 2012). The policy was intended to address the challenges and health needs of women and adolescents, particularly family planning needs (Department of Social Development 2015; Patel 2014).

Implanon NXT was introduced into the South African national contraception programme in early 2014 to increase the range of contraceptive options for women, particularly long-acting methods (Duvall et al. 2014). Implanon NXT is a single rod sub-dermal contraceptive implant that contains 68 mg etonogestrel to offer contraception for 3 years (Pillay et al. 2017). The method is long acting, reversible and not dependent on user adherence for effectiveness (Henry et al. 2014). The implant was received with much anticipation, with almost a million women opting for the method (Pleaner et al. 2017:933). However, there has been a sharp decline in the implant uptake because of various problems encountered that resulted in most women discontinuing the method before the prescribed 3-year period lapsed (Moodley & Mahomed 2016; Pillay et al. 2017).

Among the cited reasons for discontinuation are the experienced side effects, health concerns, and desire to become pregnant and partner opposition (Burusie 2013; Siyoum et al. 2017). In the United States of America (USA), because of unpleasant side effects such as hair loss, hirsutism, weight loss, dizziness and headaches, 60% of women of childbearing age discontinued Implanon NXT before its stipulated time (Ramdhan et al. 2018). In Malaysia, 70% of women on Implanon NXT discontinued its use because of irregular menses such as prolonged bleeding and infrequent bleeding (Mastor, Khaing & Omar 2014). In South Africa, 67.3% of users removed the Implanon NXT in the first 12 months of use while 94.4% removed Implanon NXT after 24 months of insertion (Mrwebi et al. 2018). Discontinuation is not unique to South Africa. In Ethiopia, the Implanon NXT discontinuation rate was reportedly at almost 40% (Medhin et al. 2016).

Although Implanon NXT was introduced as part of a contraceptive programme as a long-acting method of contraception in South Africa to assist women of childbearing age with fewer visits to the clinic and the reduction of unwanted pregnancies (Pillay et al. 2017), because of the reported side effects, the rate of discontinuation is alarming. The implications of this include unplanned pregnancies and unwanted births, and termination of pregnancies (Asaye, Nigussie & Ambaw 2017). Therefore, this article sought to explore the experiences of women regarding the early removal of Implanon NXT in the City of Johannesburg, Region E clinics in the Gauteng province.

## Problem statement

The Implanon NXT contraceptive method was introduced as part of a contraceptive programme as a long-acting method of contraception in South African health system in 2014, to assist women of childbearing age with less visits to the clinic and reduce unwanted pregnancies (Pillay et al. 2017). According to Lince-Deroche et al. (2016), there has been a great concern regarding a sharp decline in the implant method uptake and increasing number of women returning for early removal of the implant, with intolerable side-effects cited as the primary reason. The appropriateness and sustainability of the method have thus been questioned in the country because of experienced implant side-effects that received negative attention from the general populace (including healthcare workers and the media) in South Africa.

In the studies conducted in sub-Saharan Africa, Teunissen, Grimm and Roumen (2014) indicated that between 80% and 85% of users continued the method 1 year after insertion, including women ˂ 25 years of age (Diedrich, Klein & Peipert 2014). Reduced uptake and early removal of Implanon NXT before its stipulated time were also noted in Johannesburg, Region E clinics. It is in the light of these challenges that the study sought to explore the shortcomings of the method as well as to remedy the possible gaps in the provided services.

## Purpose of the study

The purpose of the study was to explore and describe the experiences of women regarding early removal of Implanon NXT in Johannesburg Region E-clinics in Gauteng province, South Africa to gain understanding of the reasons for early removal of the device.

## Research methods and design

The study explored women’s experiences regarding early removal of Implanon NXT. A qualitative and descriptive design was followed that utilised semi-structured face-to-face interviews with open-ended questions to explore participants’ experiences. The design was appropriate as it allowed the researcher to interact and encourage the participants to expand or elaborate on their given answers and go into detail on certain points raised. The design was concerned with establishing answers to the *why’s* and *how’s* of the phenomenon in question (Pritha 2020).

### Setting of the study

The study was conducted in Johannesburg city clinics, Region E. Region E sub-district has a total number of eight clinics with the distance of 1.6 km – 6 km apart. From the eight clinics, only four clinics were chosen as data collection sites as they had a high number of clients removing Implanon NXT before the stipulated time of 3 years.

### Population

The population comprised women of all ages from the four selected clinics who had inserted Implanon NXT and had it removed before the stipulated time of 3 years. As Brick (2014) asserts, the choice was informed by an element of judgement with the intention to carefully select the study participants based on specific attributes of interest to the researcher. The purposive sampling technique is based on the researcher’s judgement on the sample (Gray, Grove & Sutherland 2017; Moule & Goodman 2014). The non-probability purposive sampling strategy was used to recruit women of childbearing age who discontinued Implanon NXT before 3 years had lapsed between 2018 and 2021. Participant recruitment was done through assistance of the clinic managers, and participants were chosen from the family planning registers from the clinics.

### Participants’ recruitment and sampling

The Clinic Manager assisted the researcher to recruit the participants. The participants were chosen from the family planning register with the assistance of the Clinic Manager. Women who were recorded in the family planning register to have the Implanon NXT removed before 3 years had elapsed were contacted and were explained about the study. The researcher then visited the clinic on the scheduled day of family planning and waited in the waiting area to meet the clients who had removed the Implanon NXT before the stipulated time and switched to other methods. A total of 14 women participants were recruited; however, data saturation was reached with the 10th participant. Sampling took place throughout the process of data collection; the number of participants was determined by the increase of new ideas and sampling stopped when there was repetition of same ideas among the participants (Holloway & Wheeler 2010). According to WHO (2023), childbearing age includes women who are between ages of 15 and 49 years. It is worth noting that in this study, participants were between the age of 20 and 40 years. In addition, they needed to have used Implanon NXT and had it removed before the 3-year period.

### Data collection procedure

#### Research instrument

To test the feasibility of the data collection tool developed, the researcher conducted a pre-test of the interview guide prior to actual data collection process commenced. From the results of pre-testing, the interview guide was modified accordingly with some of the questions restructured (Holloway & Wheeler 2010). The data collection tool was pre-tested with two women from one of the settings, and the data were subsequently not included in the results of the main study.

#### Data collection

Individual face-to-face interviews using a semi-structured guide were used to collect data. The interviews were conducted at a time convenient for each participant. The interviews were conducted from February 2021 to May 2021, and each interview lasted between 30 min and 45 min per participant. The interviews were conducted in English, IsiZulu and Setswana to accommodate participants who did not understand English. Field notes were taken, and an audio tape was used to record the conversation during the interviews with the permission of the participants. The facility managers provided a private office to conduct the interviews.

### Data analysis

Data analysis is the organisation, reduction, and transformation of data into meaning (Polit & Beck 2017). In this study, themes and sub-themes were identified from the transcribed and translated data from IsiZulu and Setswana to English. The collected data were in the form of text from field notes that were taken during the interviews and the audio recorded data. The raw data and the audio data were then handed to experts in Setswana and IsiZulu languages for verification, truthfulness and interpretation of transcripts. An experienced qualitative data analysis specialist confirmed the themes and sub-themes that emerged through a consensus meeting. The audio recordings, field notes and transcriptions were all kept secure in a password-encrypted file. The researcher manually coded according to Datt and Chetty’s (2016) eight steps of content analysis coding.

### Measures to ensure trustworthiness

Trustworthiness is the mechanism of evaluating the quality and truthfulness of the research findings and the criteria (Davies & Logan 2012). This was done to ensure the truthfulness, appropriateness and meaningfulness of the data according to the five criteria of credibility, transferability, dependability, confirmability and authenticity (Polit & Beck 2017). Credibility was achieved through triangulation and member checking. To achieve transferability, a ‘dense’ description of the methodology, the participants, the context and the setting of the research study was provided. An audit trail was used to ensure dependability of what was done and of the results. Confirmability was attained when the research results represented the experiences of women who have used Implanon NXT; and by re-reading, the meanings generated from the coding. The verbatim quotes from participants’ interviews, the audio recordings, and bracketing by the researcher ensured authenticity.

### Ethical considerations

Ethical approval to conduct the study was provided by the Sefako Makgatho Health Sciences University Research Ethics Committee (No. SMUREC/H/206/2020/PG). Permission to conduct the study was granted by Research Committee of Johannesburg Health District, and the Primary Health Care Centre manager to conduct the study in the facilities.

The participants were requested to read the provided information leaflet given and ask questions for clarity. Informed consent was obtained from the participants prior to interviewing them to ensure that they were all informed about the contents of the study. The researcher read and interpreted the consent form in different languages that suited the participants, such as English, IsiZulu and Setswana, to gain their cooperation. The participants’ right to autonomy and voluntary participation was emphasised (Field & Morse, 1992 cited in Orb, Eisenhauer & Wynaden 2001).

## Results

The demographic data included participants’ age, parity, nationality, employment status and educational level (Table 1). Age, employment and education were of significance as the researcher wanted to determine if the educational level and employment status contributed to the early Implanon NXT removal.

**TABLE 1:** Demographic data of the participants.

Criterion	Category	Frequency	Percentage
Age	20–25	1	10
	26–30	3	30
	31–35	3	30
	36–40	3	30
Number of children	2	4	40
	3	5	50
	4	1	10
Marital status	Single	4	40
	Married	6	60
Country of origin	South Africa	9	90
	Malawi	1	10
Highest qualification	Secondary	9	90
	Tertiary	1	10

Participants’ ages ranged from 25 to 40 years with average age of 32.6 years. Only one participant was unemployed while the rest were employed. In terms of the highest education achieved by the participants, only 1 out of 10 had a tertiary level qualification while the remaining 9 had secondary level education.

## Summary of themes and sub-themes

A total of three themes each with two subthemes emerged and are summarised in Table 2 below.

**TABLE 2:** Themes and sub-themes.

Themes	Sub-themes
1. Positive experiences related to the use of Implanon NXT	1.1. Convenient and reliable 1.2. Efficient method for family planning
2. Negative experiences related to the use of Implanon NXT	2.1. Unpleasant effects and reasons for removal of Implanon NXT
3. The future of Implanon NXT use	3.1. Promote uptake 3.2. Discourage uptake

### Theme 1: Positive experiences related to the use of Implanon NXT

Participants indicated that they were mainly motivated to choose Implanon NXT as their method of contraception because of its benefits that included less frequent clinic visits and no need for adherence. Participants further reported on the positive experiences they had with Implanon NXT as a choice of contraceptive method.

#### Sub-theme 1.1: Convenient and reliable

The participants indicated that the method was reliable and they were happy with the benefits of using the implant. The fewer visits to the clinic was an added advantage as they did not have to be absent from work to go to the clinic. Some participants explained that the Implanon NXT was convenient to them because they did not have to worry about forgetting to take their contraceptive pills:

‘I choose Implanon NXT because I do not have to be absent at work and it stays longer [*3 years*].’ (P3, Single, 29 years)

‘… so, I decided that Implanon NXT it’s a good method for me since I forget pills and I am afraid of needles. The other reason that made me to choose it is that I will not have to visit clinic frequently.’ (P6, Single, 33 years)

Opting for Implanon NXT for the participants meant that frequent clinic visits were not required, and participants would not miss work, and those who forget pills would not have to worry about unwanted pregnancies.

#### Sub-theme 1.2: Efficient method for family planning

Some participants stated that Implanon NXT was a safe, efficient and effective family planning method as they could remove the device at any time and then fall pregnant. One participant reported that she decided to stop using the Implanon NXT because she wanted to continue with childbearing. The quote to support this is as follows:

‘My husband and I decided to have a 4th child since my children were older, so I removed it so we can try for a baby.’ (P7, Married, 39 years)

This study has identified that women perceive Implanon NXT as an effective method of contraception because of its long-acting ability. The quote in favour of this was as follows:

‘It last long, takes long to go to clinic and I was waiting for the right time to do sterilization.’ (P5, Single, 30 years)

### Theme 2: Negative experiences related to the use of Implanon NXT

Some of the participants had negative experiences regarding the use of Implanon NXT. Participants indicated that they have experienced unpleasant side effects such as heavy bleeding, inability to sexually satisfy their partners, and increased costs of having to buy the sanitary pads.

#### Sub-theme 2.1: Unpleasant effects and reasons for removal of Implanon NXT

The participants reported side effects such as abnormal bleeding patterns that included prolonged bleeding. Quotes to support this are evident in the responses below:

‘I was having heavy bleeding with clots and bleeding would stop for few days and restart again.’ (P1, Single, 34 years)

‘I cannot satisfy my husband sexually because of this bleeding.’ (P4, Married, 27 years)

‘I am bleeding almost every day and my husband is now in South Africa with me so I am afraid he will go look for women outside and he may bring me diseases.’ (P2, Married, 34 years)

Another challenge experienced by some participants was the cost of sanitary pads and, in addition, their self-esteem deteriorated as they were always bleeding. This is reflected in the following quotes:

‘My partner was no longer happy because I was always bleeding, and I was starting to feel dizzy, and it began to be costly as I was buying sanitary pads regularly.’ (P3, Single, 29 years)

‘I lost self-esteem and used lots of money buying pads.’ (P9, Married, 28 years)

‘The bleeding was becoming too much for me and I was running out of cash to buy sanitary pads.’ (P4, Married, 27 years)

One participant indicated that because of cultural beliefs about menstruation she had to stop using the Implanon NXT because she was no longer having her periods:

‘I stopped seeing my periods and it made me as you know that in our culture the pride of a woman is menstruation.’ (P5, Single, 30 years)

Another participant added:

‘I never saw my periods since I inserted Implanon NXT and now of late my skin colour is changing I am becoming dark, and I am also gaining weight. I think it is Implanon NXT that is making me have this problem.’ (P8, Married, 37 years)

One participant shared that when she was started on chronic medication her Implanon NXT was removed because the treatment she was given would make it weak. The quote to support this is as follows:

‘I found out that I am HIV [*human immunodeficiency viruses*] positive and the nurse who was helping me told me that the antiretroviral [*ARVs*] that she is giving me will make the Implanon NXT weak and I could fall pregnant on Implanon NXT.’ (P6, Single, 33 years)

### Theme 3: The future use of Implanon NXT

The willingness of the women to continue using Implanon NXT or recommending it to others will determine its future use. The study findings under this theme are rather ambiguous as different opposing views were shared by the participants.

The findings in this study also revealed that women would still recommend Implanon NXT to other women irrespective of their negative experiences because they believed that women respond differently to medication. The participants found that the reduced number of clinical visits were beneficial as it prevented absence from work. The quote to support this is as follows:

‘Yes, I can encourage someone to use it because if you are working you do not have to go to clinic often. It was minus one problem for me when I did not see my period because I did not have to buy sanitary pads so I would recommend it.’ (P7, Married, 39 years)

On the contrary, some of the participants indicated that they would not recommend the Implanon NXT to other women. The participants said:

‘No, I think they will have the same problem I had.’ (P10, Single, 23 years)

‘No, I will not recommend it because if they test positive for HIV [*human immunodeficiency viruses*] and they are told that Implanon NXT is not working well with ARVs they could be pregnant as one type of ARV that I was using was making Implanon NXT weak.’ (P8, Married, 37 years)

## Discussion

The study aimed to explore and describe the experiences of women regarding early removal of Implanon NXT. The key themes identified relate to both the positive and negative experiences of women regarding Implanon NXT, as well as uncertainty of the future of the implant. Various reasons shared by participants for the implant use included no need for adherence, and prevention of unwanted pregnancy for long periods. Many participants cited the convenience of infrequent clinic visits as they were in tertiary education; and their class attendace was not affected.

The study findings corroborate those of Pillay et al. (2017) who also stated that the motivations for the implant use were strongly tied to the method’s convenience, such as its long action and lack of requirement for adherence by the user. Reductions in clinic visits were especially important for women working or studying. In addition, some women described their experiences as ‘eye-opening’ and ‘life-changing’ secondary to fewer or lighter periods, which were viewed as a positive experience, and they furthermore enjoyed the convenience as they saved on the cost of sanitary products (Inoue et al. 2016).

Participants were also content with being able to plan for pregnancy and child spacing while having the implant. The findings were consistent with literature from Ethiopia (Asaye et al. 2017) and South Africa (Mrwebi et al. 2018; Nageso & Gebretsadik 2018), where some women reported the desire to fall pregnant as the reason for the removal of Implanon NXT. Additional benefits of Implanon NXT are that there is the rapid return to a normal menstrual cycle and no delay in returning to fertility after removal (Blumenthal, Voedisch & Gemzell-Danielsson 2012). Implanon NXT is considered the most effective long-acting reversible contraceptive used widely in sub-Saharan Africa (Thomas 2021). It is worth noting that the contraceptive’s effectiveness and dissatisfaction with other methods were the main reasons why some women opted for the implant as a method of contraception.

While some women enjoyed the positive experiences, other women removed and/or discontinued the implant before the due date. The major reasons for removing the implant prematurely were side effects, which included prolonged and irregular bleeding. The findings are consistent with those of Ojule (2016) and Tadesse et al. (2017). Interestingly, one woman cited cultural reasons for discontinuing the implant. The woman wanted to have her monthly menstrual period to earn her respect as a woman. To some extent, sexual partners also had an influence on women removing the implant, as it affected their relations, especially when prolonged bleeding was experienced.

The implant removals were mostly common among women who were cohabiting and those who were married. Similar disorders were reported as some of the factors associated with discontinuation of the Implanon NXT among women (Akilimali et al. 2020). Therefore, it can be deduced that prolonged bleeding is a common negative effect experienced by women that puts strain on their sexual relations. There is a need for women who choose Implanon NXT to be given pre-insertion counselling so that they are well informed about the side effects, as this might assist them to deal with the side effects and therefore increase their chances of continuing the method.

Some women stated that their self-esteem and quality of life were affected. They carried anxiety and fear of getting their underwear stained with blood from the heavy bleeding (Gokyildiz et al. 2013). Studies conducted in Nigeria and South Africa revealed that the high discontinuation rate of the implant was reportedly because of side effects such as bleeding irregularities, acne, mood changes and the desire to fall pregnant (Balogun et al. 2014; Mrwebi et al. 2018). The discontinuation of Implanon is generally mostly associated with bleeding pattern disruption (WHO 2015).

Drug interaction with chronic medication was also cited as a reason for discontinuation of the implant. It has been reported that chronic drugs such as ARVs compromise Implanon NXT’s efficacy (Patel et al. 2018; Robinson, Jamshidi & Burke 2012). Women were encouraged to use dual protection to prevent unwanted pregnancy and the spread of infections (Pillay et al. 2017:813).

Contrastingly, some of the participants reported that they would recommend Implanon NXT to other women, while others reported that they would not recommend the method to other women. A study conducted to perform a direct comparison of the satisfaction of intrauterine device users and the Implanon NXT users indicated that after 6 months women were reported to be less likely to recommend these methods to others because of the dissatisfaction they experienced with both the methods (Wong et al. 2010). However, like the findings in this study, other studies also found that Implanon NXT users, even those with negative experiences, would recommend the method to the other women (Howett et al. 2021).

## Conclusion and recommendations

The study findings indicated that heavy and irregular bleeding was the main reason for Implanon NXT discontinuation. The need to have more children and interference with sexual life were also a concern. The findings of the study suggest that proper pre-counselling services be provided to improve women’s education before implant insertion. This could assist the potential users to make an informed decision regarding the appropriateness of the method for them. Furthermore, counselling about the side effects should be done on a continuous basis to prepare the users as some women experience the side effects later on. Moreover, knowledge on how to manage the side effects would encourage clients to keep Implanon NXT for the expected duration of 3 years. It is therefore crucial that the health care providers are afforded training opportunities in order to provide quality contraceptive health services; and for health promoters to ensure the implementation of the policy as this will inform clients’ expanded choice, planning and decisions-making.

## Limitations

All participants were drawn from only four clinics out of the eight clinics in Region E; therefore, the findings of the study could not be generalised as this is not a representative sample.

## References

[CIT0001] Akilimali, P.Z., Hernandez, P., Angelwicz, P., Kayembe, K. & Bertrand, J., 2020, ‘Cumulative discontinuation rate over time’, *Journal for Reproductive Health* 2(1), 34–45.

[CIT0002] Asaye, M., Nigussie, T. & Ambaw, W., 2017, ‘Early Implanon discontinuation and associated factors among Implanon user women’, in Debre Tabor Town’, *International Journal of Reproductive Medicine* 7(1), 6–11. 10.1155/2018/3597487 PMC5896376 29796392

[CIT0003] Balogun, O.R., Olaomo, N., Adeniran, A.S. & Fawole, A.A., 2014, ‘Implanon sub-dermal implant: An emerging method of contraception in Ilorin, Nigeria’, *Journal of Medical and Biomedical Sciences* 3(1), 1–5. 10.4314/jmbs.v3i1.1

[CIT0004] Blumenthal, P.D., Voedisch, A. & Gemzell-Danielsson, K., 2011, ‘Strategies to prevent unintended pregnancy: Increasing use of long-acting reversible contraception’, *Human Reproduction Update* 17(1), 121–137. 10.1093/humupd/dmq026 20634208

[CIT0005] Brick, J.M., 2014, *On making inferences from non-probability samples*, Washington Statistical Society, President’s Invited Seminar, Washington, DC.

[CIT0006] Burusie, A., 2013, ‘Reasons for premature removal of Implanon among users in Arsi zone, Oromia region, Ethiopia’, *Journal for Health Sciences* 4(1), 132–141.

[CIT0007] Datt, S. & Chetty, P., 2016, *8 steps procedure to conduct qualitative content analysis in research*, 3rd edn., Sage, New York, NY.

[CIT0008] Davies, B. & Logan, J., 2012, *Reading research: A user-friendly guide for health professionals*, 5th edn., Elsevier, Toronto.

[CIT0009] Department of Social Development, 2015, *National adolescent sexual and reproductive health and rights framework strategy, 2014–2019*, DSD, Pretoria.

[CIT0010] Diedrich, J.T., Klein, D.A. & Peipert, J.F., 2017, ‘Long-acting reversible contraception in adolescents: A systematic review and meta-analysis’, *American Journal of Obstetrics and Gynecology* 216(4), ee1–e64. 10.1016/j.ajog.2016.12.024 28038902

[CIT0011] Duvall, S., Thurston, S., Weinberger, M., Nuccio, O. Montgomery, N.F., 2014, ‘Scaling up delivery of contraceptive implants in sub-Saharan Africa: Operational experiences of Marie Stopes International’, *Global Health: Science and Practice* 2(1), 72–92. 10.9745/GHSP-D-13-00116 25276564 PMC4168608

[CIT0012] Gokyildiz, S., Aslan, E., Beji, N.K. & MeCdi, M., 2013, ‘The effects of menorrhagia on women’s quality of life: A case-control study’, *International Scholarly Research Notices* 91, 73–79. 10.1155/2013/918179 PMC3734607 23970973

[CIT0013] Gray, J.R., Grove, S.K. & Sutherland, S., 2017, *The practice of nursing research: Appraisal, synthesis, and generation of evidence*, 8th edn., Elsevier, St Louis.

[CIT0014] Henry, N., Schlueter, M., Lowin, J., Lekander, I., Filonenko, A., Trussell, J. et al., ‘Cost of unintended pregnancy in Norway: A role for long-acting reversible contraception’, *Journal for Family Planning Reproductive Health Care* 41(2), 10–15. 10.1136/jfprhc-2014-100878 PMC4369438 25537792

[CIT0015] Holloway, I. & Wheeler, S., 2010, *Qualitative research in nursing and healthcare*, 3rd edn., Wiley-Blackwell, Chichester.

[CIT0016] Howett, R., Krogstad, E.A., Badubi, O., Gertz, A.M., Bawn, C., Mussa, A. et al., 2021, ‘Experiences of accessing and providing contraceptive implant removal services in Gaborone, Botswana: A qualitative study among implant users and healthcare providers. Global Women’s Health (online)’, *Frontiers in Global Women’s Health* 2, 684694. 10.3389/fgwh.2021.684694 PMC8593984 34816231

[CIT0017] Inoue, K., Kelly, M., Barratt, A., Bateson, D., Rutherford, A., Black, K.I. et al., 2016, ‘Australian women’s experience of the subdermal contraceptive implant: A qualitative perspective’, *Journal for Australian Family Physician* 45(2), 734–739. 27695724

[CIT0018] Lince-Deroche, N., Pleaner, M., Morroni, C., Mullick, S., Firnhaber, C., Harries, J. et al., 2016, ‘Achieving universal access to sexual and reproductive health services: The potential and pitfalls for contraceptive services in South Africa’, *South African Health Review* 2016(1), 95–108.

[CIT0019] Mastor, A., Khaing, C. & Omar, S., 2014, ‘User’s perspective on Implanon’, *Journal of Contraception* 2(2), 5–7.

[CIT0020] Medhin, T., Gebrekidan, K., Nerea, M., Gerezgiher, H. & Haftu, M., 2016, ‘Early Implanon discontinuation rate and its associated factors in health institutions. Department of Midwifery’, *Journal of Sexual and Reproductive Health* 1(1), 30–32.

[CIT0021] Moodley, A. & Mahomed, O., 2016, ‘Prevalence and predictors of Implanon uptake in Ugu’, *Journal for South African Family Practice* 61(2), 7–8. 10.4102/safp.v61i2.5006

[CIT0022] Moule, P. & Goodman, M., 2014, *Nursing research*, SAGE, London.

[CIT0023] Mrwebi, P., Ter Goon, D., Eyitayo, O., Oladele, V., Seekoe, E. & Ajayi, A., 2018, ‘Reasons for discontinuation of Implanon among users in Buffalo City Metropolitan’, *African Journal of Reproductive Health* 22(1), 118–126. 10.29063/ajrh2018/v22i1.11 29777648

[CIT0024] Nageso, A. & Gebretsadik, A., 2018, ‘Discontinuation rate of Implanon and its associated factors among women who ever used Implanon in Dale District, Southern Ethiopia. BMC Women’s Health’, *BMC Women’s Health* 18(1), 189. 10.1186/s12905-018-0678-x 30453931 PMC6245529

[CIT0025] National Adolescent Sexual and Reproductive Health and Rights Framework Strategy, 2014–2019, *National Department of Health*, viewed 09 September 2022, from http://www.dsd.gov.za/index2.php?option=com_docman&task=doc_view&gid=578&Itemid%E2%80%A6=.

[CIT0026] National Contraceptive Guidelines in South Africa, 2012, *A companion to the National Contraception and Fertility Planning Policy and Service Delivery Guidelines*, Department of Health, Pretoria.

[CIT0027] Ojule, J.D., 2018, ‘Implanon implant contraception at the university of Port Harcourt teaching hospital’, *Asian Journal of Medicine and Health* 11, 1–6. 10.9734/AJMAH/2018/40017

[CIT0028] Orb, A., Eisenhauer, L. & Wynaden, D., 2001, ‘Ethics in qualitative research’, *Journal of Nursing Scholarship* 33, 93–96. 10.1111/j.1547-5069.2001.00093.x 11253591

[CIT0029] Patel, M., 2014, ‘Contraception: Everyone’s responsibility’, *South African Medical Journal* 104(9), 644. 10.7196/SAMJ.8764 26307790

[CIT0030] Patel, R., Stalter, R., Thomas, K., Tamar, B., Blue, W., Erikson, D. et al., 2019, ‘Concomitant contraceptive implant and Efavirenz use in women living with HIV: Perspectives on current evidence and policy implications for family planning and HIV treatment guidelines’, *Journal for International AIDS Society* 20(1), 12–17. 10.7448/IAS.20.1.21396 PMC5515020 28530033

[CIT0031] Pillay, D., Chersich, M., Morroni, C., Pleaser, M., Adeagbo, O., Naidoo, N. et al., ‘User perspectives on Implanon NXT in South Africa: A survey of 12 public sector facilities’, *South African Medical Journal* 107(10), 45–49. 10.7196/SAMJ.2017.v107i10.12833 29397680

[CIT0032] Pleaner, M., Morroni, C., Smit, J., Lince-Deroche, N., Chersich, M.F. & Mullick, S., 2017, ‘Lessons learnt from the introduction of the contraceptive implant in South Africa’, *South African Medical Journal* 107(11), 933–938. 10.7196/SAMJ.2017.v107i11.12805 29399422

[CIT0033] Polit, D.F. & Beck, C.T., 2017, *Nursing research: Generating and assessing evidence for nursing practice*, 10th edn., Wolters Kluwer, Philadelphia, PA.

[CIT0034] Pritha, B., 2020, ‘Introduction to qualitative research’, *Journal for Community Medicine* 1(3), 21–29.

[CIT0035] Ramdhan, C., Simonds, E., Wilson, C., Loukas, M., Oskouian, R. & Tubbs, S., 2018, ‘Complications of subcutaneous contraception’, *Journal for Medicine* 10(1), 34–38. 10.7759/cureus.2132 PMC5878093 29610715

[CIT0036] Robinson, J.A., Jamshidi, R. & Burke, A.E., 2012, ‘Contraception and antiretroviral therapy’, *Journal for Infectious Diseases in Obstetrics and Gynaecology* 2, 31–36. 10.1155/2012/890160 PMC3426212 22927715

[CIT0037] RSA (Republic of South Africa), 1996, *The Constitution of the Republic of South Africa. Act 108 of 1996*, viewed 12 June 2022, from http://www.gov.za/DOCUMENTS/CONSTITUTION/constitution-republic-south-africa-1996-1.

[CIT0038] Siyoum, M., Mulaw, Z., Abuhay, M. & Kebebe, H., 2017, ‘Implanon discontinuation and associated factors among women who ever used Implanon’, *Journal of Public Health and Community Medicine* 4(1), 3–5.

[CIT0039] Stover, J. & Ross, J., 2010, ‘How increased contraceptive use has reduced maternal mortality’, *Maternal Child Health Journal* 14, 687–695. 10.1007/s10995-009-0505-y 19644742

[CIT0040] Tadesse, A., Kondale, M., Agedew, E., Gebremeskel, F., Boti, N. & Oumer, B., 2017, ‘Determinant of Implanon discontinuation among women who ever used Implanon in Diguna Fango District, Wolayita Zone, Southern Ethiopia: A community-based case control study’, *International Journal of Reproductive Medicne* 2017(1), 2–4. 10.1155/2017/2861207.22 PMC5695023 29234726

[CIT0041] Teunissen, A.M., Grimm, B. & Roumen, F.J., 2014, ‘Continuation rates of the subdermal contraceptive Implanon^®^ and associated influencing factors’, *The European Journal of Contraception & Reproductive Health Care* 19(1), 15–21. 10.3109/13625187.2013.862231 24329119

[CIT0042] Thomas, L., 2021, ‘Advantages and disadvantages of contraceptive implants’, *News-medical*, viewed 25 May 2022, from https://www.news-medical.net/health/advantages-anddisadvantages-of-the-contraceptive-implant.aspx.

[CIT0043] United Nations Department of Economic and Social Affairs, 2015, *Sustainable Development Goals: Sustainable Development Knowledge Platform*, UNDESA, Geneva.

[CIT0044] Wong, R., Bell, R.J., Kalyani, T., McNamee, M. & Vollenhoven, B.J., 2010, ‘Implanon users are less likely to be satisfied with their contraception after six months than IUD users’, *Contribution Journal* 80(5), 452–456. 10.1016/j.contraception.2009.03.021 19835719

[CIT0045] World Health Organization (WHO), 2015, *Department of reproductive health and research. WHO statement on progestogen-only implants*, World Health Organization, Geneva.

[CIT0046] World Health Organization (WHO), 2020, *Fact sheet. Family planning/contraception methods*, World Health Organization, Geneva.

